# Editorial: Systems Biology and Ecology of Microbial Mat Communities

**DOI:** 10.3389/fmicb.2016.00115

**Published:** 2016-02-09

**Authors:** Martin G. Klotz, Donald A. Bryant, Jim K. Fredrickson, William P. Inskeep, Michael Kühl

**Affiliations:** ^1^Department of Biology, Queens College of The City University of New YorkNew York, NY, USA; ^2^Department of Biochemistry and Molecular Biology, The Pennsylvania State UniversityUniversity Park, PA, USA; ^3^Department of Chemistry and Biochemistry, Montana State UniversityBozeman, MT, USA; ^4^Pacific Northwest National LaboratoryRichland, WA, USA; ^5^Department of Land Resources and Environmental Sciences, Montana State UniversityBozeman, MT, USA; ^6^Department of Biology, University of CopenhagenHelsingør, Denmark

**Keywords:** chemotrophy, diel cycling, metabolomics, metagenomics, microbial mats, microsensors, photosynthesis, proteomics

The goals of systems biology and microbial ecology are to gain a predictive understanding of how microbial communities function in dynamic systems, inclusive of interactions among community members and spatiotemporal changes in the physicochemical environment. Microorganisms in natural systems experience cycles of environmental change over different periodicities and amplitudes, and these processes are reflected in the composition, genetic repertoire and activity of microbial community members, and community function as a whole. A primary emphasis of this research topic was to focus on reports of tractable microbial communities in extreme environments (e.g., temperature, salinity, light, pH) where a foundation of genome sequence and other molecular (-omic) and geochemical measurements provide evidence of specific functional attributes of individual community members, which are directly linked with spatiotemporal changes in key environmental variables as well as the metabolic dynamics of other community members. Thermal or saline microbial mats are often stratified with respect to key environmental variables (e.g., light, oxygen) and exhibit compositional simplicity and low heterogeneity relative to habitats such as soils and/or natural waters. Consequently, the majority of contributions to this research topic focus on either high-temperature chemotrophic microbial mats, high-temperature phototrophic mats, or hypersaline phototrophic mats in marine or epsomitic systems. The structure and function of high-temperature systems of Yellowstone National Park (YNP) was evaluated using metagenome sequence and geochemical observations across a wide range of environmental conditions, which provided a basis for understanding the distribution of thermophiles in YNP and led to the discovery of several new archaeal and bacterial lineages. Genome sequences of relevant ecotypes have provided a foundation for interrogating more detailed spatiotemporal aspects of thermophilic phototrophic communities, including microsensor analysis of their physical and chemical microenvironment, and provided a rationale for comparison to hypersaline phototrophic mats. The fixation of carbon dioxide as a primary carbon source and the production of key cofactors by autotrophs are important processes, which support diverse heterotrophs across widely different environmental circumstances. Specific metabolic linkages among community members (e.g., production of storage compounds, nitrogen fixation, fermentation, sulfate reduction, hydrogen, and vitamin production) were documented in phototrophic mats, which revealed that these processes changed in unexpected ways across a diel cycle. The tight metabolic coupling among specific populations that are highly adapted to spatial and/or temporal conditions is a common theme in natural environments, and this was demonstrated in detail using different light-adapted ecotypes of cyanobacteria (*Synechococcus* spp.) present in alkaline siliceous geothermal mats as primary producers. Finally, controlled experiments using pure cultures and/or consortia as “systems” revealed the importance of specific nutrient requirements, and importantly, how the exoproteome of a bacterium is controlled by the level of cellular oxidation. Ultimately, a predictive understanding of the complex network of abiotic and biotic interactions that occur in natural systems will also require detailed knowledge of the regulatory and physicochemical processes that govern gene expression, post-translational modifications, and protein activity. It is our hope that the articles included in this research topic advance a more comprehensive understanding of natural microbial communities, and demonstrate the utility of coupling molecular methods with detailed spatiotemporal measurements and dissection of microbial community function across gradients in key environmental variables such as light, temperature, pH, oxygen, or hydrogen. Integrated approaches across relevant microbial scales will lead to predictive capabilities useful for engineering microbial communities (or consortia) and for understanding how natural systems may respond to changes in key environmental variables (e.g., climate change).

## Author contributions

WI drafted the manuscript, DB, JF, MK, and MGK revised the draft and all authors agreed to the final version. The articles in the RT were edited by MGK (10), WI (3), MK (1), and DB (1).

### Conflict of interest statement

The authors declare that the research was conducted in the absence of any commercial or financial relationships that could be construed as a potential conflict of interest.

